# Taxonomic and functional diversity of alkali-tolerant bacteria enriched from the Taklimakan Desert

**DOI:** 10.3389/fmicb.2025.1580401

**Published:** 2025-08-07

**Authors:** Feng Wen, Guo Yang, Xueling Zhao, Tiantian Zhao, Linquan Bai, Zhanfeng Xia

**Affiliations:** ^1^Key Laboratory of Protection and Utilization of Biological Resources in Tarim Basin, College of Life Sciences and Technology, Xinjiang Production & Construction Corps, Tarim University, Alar, China; ^2^State Key Laboratory of Microbial Metabolism, Shanghai-Islamabad-Belgrade Joint Innovation Center on Antibacterial Resistances, School of Life Sciences and Biotechnology, Shanghai Jiao Tong University, Shanghai, China

**Keywords:** Taklimakan Desert, alkali-resistant microorganisms, amylase, protease, cellulase

## Abstract

The Taklimakan Desert is a naturally alkaline ecosystem harboring a rich diversity of alkali-resistant microorganisms. However, systematic studies on their distribution, diversity, and biotechnological potential remain limited. In this study, five representative soil samples were collected from the central region of the Taklimakan Desert, where the original soil pH ranged from 8.78 to 9.8. To investigate the effect of alkaline conditions on microbial communities, the samples were subjected to enrichment in culture media adjusted to pH 9–11. The bacterial community structure of the enriched fraction was assessed using culture-independent 16S rRNA gene amplicon sequencing, while a non-enriched control (CK) group—consisting of the same soils without pH adjustment—was simultaneously sequenced to determine the baseline bacterial composition. In parallel, a culture-dependent approach was employed to isolate alkali-tolerant bacterial strains from the same samples using Gibbons medium at pH 9, 10, and 11. Based on distinct colony morphologies, isolates were selected, repeatedly purified by streaking, and taxonomically identified via 16S rRNA gene sequencing, resulting in a total of 291 strains. These isolates were taxonomically assigned to four phyla, six classes, 17 orders, 25 families, and 56 genera. Among them, 114 strains shared less than 98.65% sequence identity with known species, suggesting the presence of numerous potential novel taxa. Approximately 14.07 and 61.48% of the isolates were categorized as alkali-tolerant and alkalophilic, respectively, with 85 strains capable of growing under extreme conditions (pH 12 and/or 25% salinity). Functional screening revealed enzymatic activity in a substantial portion of the isolates: 20.35% produced amylase, 19.91% protease, 30.30% cellulase, and 47.61% exhibited at least one enzymatic function. Overall, this study integrates both culture-independent and culture-dependent approaches to reveal the taxonomic and functional diversity of alkali-tolerant bacteria in the Taklimakan Desert, highlighting their ecological roles and potential applications in industrial biotechnology.

## Graphical abstract

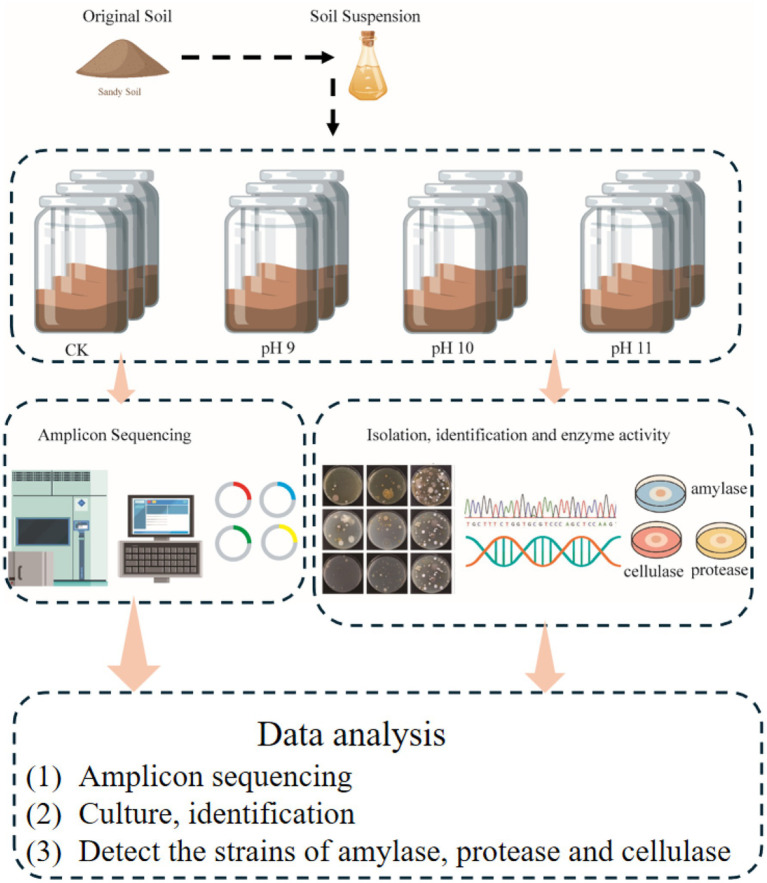

## 1 Introduction

Extreme environments possess unique ecological characteristics, such as high or low temperatures, high pressures, high salt concentrations, and the superimposition of two or more extreme factors (Cene et al., 2023). Organisms that live in extreme environments are called extremophiles. Desert environments, due to their distinctive geological and climatic conditions, are typical extreme environments. As the second largest mobile desert in the world, the Taklimakan Desert is characterized by extreme aridity, nutrient deficiency, high temperatures, intense solar radiation, and high salinity and alkalinity, which are unfavorable for life and are accompanied by frequent sandstorms (Becherucci et al., 2019; Gu et al., 2017; Santiago et al., 2018), forming an extreme and complex ecosystem. These special environmental conditions have created a unique microbial ecosystem in the Taklimakan Desert, making it an important area for studying extremophiles (Wang et al., 2008). Due to the harsh environment, microorganisms living in deserts have remarkable stress resistance, enabling them to survive and reproduce under drought, high salt, and high alkaline conditions. These extremophiles have become important resources in fields such as bioengineering and biomedicine, with their stress-resistance genes and active metabolites demonstrating significant research and application value (Abdul et al., 2018; Cowan et al., 2020; Meng et al., 2019; Perera et al., 2018). The salinized soil and high alkalinity of the Taklimakan Desert have resulted in the emergence of numerous alkaliphilic and halotolerant microorganisms with high stress resistance and rich species diversity.

In addition to extreme aridity and temperature fluctuations, desert ecosystems are often characterized by high salinity and alkalinity—two abiotic stressors that pose significant challenges to microbial survival and functionality. In particular, the Taklimakan Desert exhibits both intense salinization and persistent alkaline conditions due to its geological history, minimal precipitation, and high evaporation rates. These stresses can disrupt cellular homeostasis by affecting protein structure, enzyme activity, membrane integrity, and nutrient availability.

According to ecological theory, long-term exposure to these stressors exerts strong selective pressure on soil microbial communities, favoring the survival and proliferation of extremotolerant taxa such as alkali-tolerant and salt-tolerant bacteria. These organisms have evolved not only structural and physiological adaptations but also functional strategies—such as the secretion of extracellular hydrolytic enzymes—to enhance nutrient acquisition in nutrient-poor environments. These enzymes help degrade complex organic matter, release assimilable nutrients, and support microbial colonization by promoting biofilm formation and resource partitioning. Therefore, the abundance of such bacteria in desert soils is an ecological outcome of both environmental filtering and adaptive advantage (Fierer and Jackson, 2006; Pointing and Belnap, 2012). In turn, the stability and robustness of these enzymes under high pH and salinity reflect their functional roles *in situ* and provide the basis for their industrial value (Raddadi et al., 2015; Rath et al., 2019; Zucconi et al., 2020). For example, enzymes like amylases, proteases, and cellulases produced by desert-adapted bacteria retain catalytic activity under extreme physicochemical conditions, making them highly suitable for industrial applications in detergents, food processing, and bioenergy production (Raddadi et al., 2015; Oren, 2010).

To survive under such hostile conditions, desert-dwelling microorganisms have evolved specialized adaptations. These include the synthesis of osmoprotectants (e.g., glycine betaine, ectoine), modifications to cell wall and membrane composition, and the production of stress-tolerant enzymes that retain activity under high salt or alkaline pH. Some alkaliphiles maintain cytoplasmic pH homeostasis via active ion transport mechanisms or proton extrusion systems. Others generate extracellular polymeric substances (EPS) to buffer environmental stress and facilitate biofilm formation. These adaptive strategies not only ensure microbial survival but also contribute to their ecological functionality and potential utility in biotechnology. However, systematic studies on the diversity, adaptive mechanisms, and enzymatic potential of salt-tolerant and alkali-tolerant microbes in hyperarid desert environments remain scarce.

In 1928, Downie discovered the first alkaliphile. Since then, alkaliphiles have been isolated from soil, marine, and salt-lake environments. Based on the description of Fernandez-Lopez et al. (2023), we will use the terms alkali-tolerant and basophilic in this article. Microorganisms that have long been inhabiting alkaline environments have evolved unique cellular structures, physiological functions, and genetic characteristics (Dorado et al., 2001) to adapt to alkaline conditions, demonstrating their significant application value in industry and other fields. Enzymes synthesized by alkaliphiles typically exhibit high activity and stability (Morozkina et al., 2010; Oren, 2008), showing great potential for applications in multiple fields such as life sciences, food, medicine, and new energy development. Various functional enzymes have extensive industrial applications. Proteases are widely used in contact lens cleaners, cheese production, and meat processing (Gupta et al., 2002; Sharma et al., 2017). Amylases are mainly used in the food industry (baking and juice processing), as well as in paper and textile processing, accounting for approximately 25% of the total industrial enzyme usage. Alkaline amylases remain active within the pH range of 8–11 and are used for detergent production (Sarethy et al., 2011). Alkaline cellulases are utilized for detergents used in the textile industry (Anish et al., 2010; Aziz et al., 2022).

As a naturally alkaline desert, the Taklimakan Desert harbors a large number of alkaliphilic and halotolerant microorganisms. However, systematic studies on the distribution, diversity, and application potential of alkali-resistant microorganisms in deserts are still limited. In this study, we aimed to systematically explore the diversity of alkali-tolerant and salt-tolerant bacteria in the Taklimakan Desert and evaluate their potential biotechnological applications.

To clarify the scope of our study, we performed 16S rRNA gene amplicon sequencing on enrichment cultures grown under alkaline conditions (pH 9–11), which represents the enriched fraction of potentially cultivable bacteria, rather than the total soil microbial community. A non-enriched control (CK) group consisting of the same soil samples without alkaline treatment was simultaneously sequenced to assess the baseline microbial composition.

In parallel, we isolated culturable bacterial strains using Gibbons medium at pH 9, 10, and 11, investigated their population diversity and distribution characteristics, examined their tolerance ranges to pH and NaCl, and screened them for functional enzymes including amylases, proteases, and cellulases. These findings not only enrich China’s alkaliphilic microbial and genetic resource databases but also provide a foundation for the development and utilization of alkaliphiles and stress-resistance genes, with important theoretical and practical significance.

## 2 Materials and methods

### 2.1 Samples

In July 2022, a total of 15 surface soil samples were collected from five representative locations at the eastern (Shaya County), western (Qiemo County), southern (Yutian County), northern (Alar City), and central (Lop County) margins of the Tarim Basin Desert (Table 1). At each site, three spatially independent replicate samples (0–10 cm depth) were collected within a 20 m × 20 m plot of unvegetated sandy terrain to account for microhabitat heterogeneity while ensuring environmental comparability across sites. Vegetation cover, biological crusts, and topographic depressions were deliberately avoided to minimize confounding effects. Each sample was sealed in a sterile Labplas bag (EFR-5590E; TWIRL’EM, Mississauga, ON, Canada). Subsamples were stored at −20°C for molecular analysis and at 4°C for culture-based experiments.

**Table 1:** Sample collection information.

No.	A	B	C	D	E
Direction	South	North	West	East	Central
Sampling site area	Yutian County	Alar City	Qiemo County	Shaya County	Lop County
Sample types	Sandy	Sandy	Sandy	Sandy	Sandy
Longitude	81°17′40′′E	82°5′59′′E	83°48′0′′E	82°19′41″E	80°57′25′′E
Latitude	36°54′9′′N	40°48′0′′N	39°18′50′′N	39°37′52″N	39°4′59′′N
Altitude (m)	1,364	926	990	988	1128.5
pH	8.94	9.800	9.06	8.72	9.09

All soils were collected from bare sand surfaces. For enrichment experiments, Gibbons medium was adjusted to pH 9, 10, and 11; the control group used unadjusted soil at its original pH (ranging from 8.72 to 9.8).

### 2.2 Methods

#### 2.2.1 Culture-independent experimental system construction

To investigate the response of soil microbial communities to alkaline stress, an enrichment experiment was conducted using Gibbons medium adjusted to pH 9 (P9), 10 (P10), and 11 (P11). For each treatment, 10 g of surface soil was added to 100 mL of sterile medium in a 250 mL Erlenmeyer flask. A total of five replicates were prepared per pH condition, corresponding to soil samples collected from five representative sites across the eastern (Shaya County), western (Qiemo County), southern (Yutian County), northern (Alar City), and central (Lop County) regions of the Tarim Basin Desert. The flasks were incubated in a shaking incubator at 30°C and 120 rpm for 30 days.

Following incubation, samples enriched at the same pH level (P9, P10, or P11) were pooled into a single composite sample per treatment, representing a broad alkali-tolerant bacterial community under each respective pH condition. It is important to note that DNA used for 16S rRNA gene amplicon sequencing was extracted from enrichment cultures grown under defined alkaline conditions. Therefore, the microbial profiles obtained reflect the composition of bacteria capable of growing under the specific enrichment conditions (e.g., pH, medium composition, temperature, oxygen availability), rather than the total community present in the original soil samples. The control group (CK) was composed of the same five original soil samples, and used for direct DNA extraction to reflect the baseline microbial community. This pooling strategy was employed to highlight the general microbial response to alkaline conditions across the desert ecosystem while minimizing site-specific variability.

#### 2.2.2 Soil DNA extraction and 16S rRNA amplicon-based community analysis

Total bacterial DNA was extracted from 0.5 g of each sample using the OMEGA Soil DNA Kit (M5635-02), following the manufacturer’s protocol. For the enrichment groups (P9, P10, and P11), DNA was extracted from the enriched cultures after 30 days of incubation. For the control (CK) group, DNA was directly extracted from untreated original soil samples without pH adjustment, to represent the baseline microbial community. DNA concentration was quantified using a Nanodrop spectrophotometer (Thermo Fisher Scientific, United States), and quality was assessed by 1.2% agarose gel electrophoresis.

PCR amplification of the V3–V4 region of the bacterial 16S rRNA gene was performed using primers 799F (5′-ACTCCTACGGGAGGCAGCA-3′) and 1193R (5′-GGACTACHVGGGTWTCTAAT-3′). Each 25 μL PCR reaction contained 12.5 μL of Phanta Max Master Mix, 1 μL of each primer (10 μM), 1 μL of DNA template, and 9.5 μL of ddH₂O. Thermocycling conditions were: initial denaturation at 98°C for 2 min; 25 cycles of 98°C for 15 s, 55°C for 30 s, 72°C for 30 s; followed by a final extension at 72°C for 5 min.

The PCR amplification products were sent to Shanghai Personalbio Biotechnology Co., Ltd.^1^ for sequencing. High-throughput sequencing was conducted using the Illumina NovaSeq and MiSeq platforms (Illumina, San Diego, United States). After quality control and library construction, samples were sequenced and demultiplexed based on their barcode sequences. High-quality reads were processed using the DADA2 pipeline to perform primer trimming, quality filtering, denoising, merging, and chimera removal. Amplicon sequence variants (ASVs) were assigned taxonomic identities using a feature classifier trained on the SILVA 132 reference database^2^, specifically trained for the V3–V4 region using primers 799F/1193R, via the sklearn naïve Bayes algorithm (Quast et al., 2012). All sequence processing was performed in QIIME2 (Bolyen et al., 2019), and data visualization was conducted in R version 4.3.0 (Grosboillot and Dragos, 2024).

To ensure data reliability and monitor contamination, the sequencing process included several negative controls: a DNA extraction blank (no template control during extraction), a PCR blank (no DNA template), and an autoclaved medium control to check for environmental or reagent-derived contamination. All controls were processed alongside experimental samples through library preparation and sequencing but yielded no detectable amplification products.

#### 2.2.3 Isolation and cultivation of cultivable microorganisms from the Taklimakan Desert

Gibbons separation media with final concentrations of 1, 1/5, and 1/10 were selected (Vreeland, 2018), and 1 mL of micro salt ion solution (FeSO_4_, ZnSO_4_ and MnCl_2_ at 1 g/1000 mL each) was added to 1 L of the culture medium at pH 9, 10, and 11. The purified culture medium was selected as the separation medium.

The final concentration of Gibbons isolation medium were 1, 1/5, and 1/10, and the pH was 9, 10, and 11, and 100 kPa steam sterilization was performed for 30 min. The medium was slowly shaken and poured into a sterile Petri dish, and three replicates were used for each concentration. The specific operation is to wrap sterile cotton in a sterile velvet cloth to form a similar “culture dish” shape, so that it has the same size bottom as the medium, dip about 0.1 g of soil sample, and replicate it evenly on the surface of the medium plate to separate and count culturable bacteria. It was cultured in an inverted incubator at 28°C for 14 days. The types and quantities of colonies formed over time were observed and recorded, and a single-colony purification culture was performed.

#### 2.2.4 Identification of microbial species

Alkalophilic bacteria from desert soil samples were isolated and cultured. According to the morphology, color, size and other characteristics of the colonies, representative colonies were selected for pure culture, duplicate strains were screened out. The total genomic DNA of bacterial isolates was extracted using a bacterial DNA extraction kit (Omega Bio-Tek, Norcross, GA, USA). The 16S rRNA genes of the purified strains were amplified using universal bacterial primers Eubac 27F (5′-AGAGTTTGATCCTGGCTCAG-3′) and Eubac 1492R (5′-TACGGYTACCTTGTTACGACTT-3′) as described by Ludwig (2007). The PCR products were then evaluated by electrophoresis on a 1% agarose gel at 120 V for 30 min to confirm amplification quality. Electrophoresis results were recorded using a gel imager, and PCR products with clear, bright bands of appropriate sizes were selected and sent to the sequencing companies. Sequencing results were analyzed using SeqMan software Pro V 7.1.0 to splice the base sequences in EzBioCloud^3^ (Yoon et al., 2017), compare them to those in the database, and determine the microbial species relationship. The phylogenetic tree was constructed using MEGA 6.0 (Tamura et al., 2013) to infer the phylogenetic relationships among the isolated strains, which reflects one aspect of their taxonomic placement. ClustalW (Larkin et al., 2007) was used to compare sequences. Evolutionary distances were calculated using Kimura’s two-parameter model (Nishimaki and Sato, 2019). Bootstrapping with 1000 resamples was used to calculate the confidence values of the evolutionary tree topology (Felsenstein, 1985).

#### 2.2.5 Culture preservation

Glycerol tube cryopreservation and vacuum freeze-drying preservation methods (Om et al., 2013) were used to preserve the isolated strains.

#### 2.2.6 Strain nucleotide sequence entry number

Among the 291 isolates identified, the 16S rRNA gene sequences of 271 non-duplicate isolates were selected and submitted to the GenBank database with entry numbers PQ825971-PQ826240.

#### 2.2.7 Determination of the pH tolerance range

With Gibbons medium as the base medium, the adjusted pH values were 9, 10, 11, and 12. The 270 non-duplicated bacterial strains were inoculated on medium plates with different alkalinity and cultured at 30°C for 7 days. Whether the strains grew and were resistant to the corresponding alkalinity was determined, and the strains with strong alkali resistance were screened.

#### 2.2.8 Determination of the salinity tolerance range

To determine the salinity tolerance of the strains, the salinity of the Gibbons culture medium was continuously increased. NaCl was added at 0, 5, 10, 15, 20, and 25%, while the pH of the Gibbons culture medium maintained at 9.0. The strains were inoculated on medium plates with different salinity and cultured at 30°C for 7 days to determine whether the strain was tolerant to the corresponding salinity according to whether the area of the strain grew, in order to identify the strains with strong salt tolerance.

#### 2.2.9 Determination of the high salt and alkali resistance range

With the medium maintained at pH 12, 15 and 25% NaCl was added. The strain was inoculated on the medium plate with high salinity, the growth of the strain was tested in the medium with 15 and 25% salinity, and the strains were cultured at 30°C for 7 days to determine their tolerance for high salinity based on whether the strain grew, in order to identify strains with high salinity tolerance.

#### 2.2.10 Enzyme activity screening

The screening media for enzyme activities were prepared as follows. The amylase activity assay medium contained (per liter): 10 g soluble starch, 0.3 g MgCO₃, 1.0 g KNO₃, 0.5 g NaCl, 3.0 g K₂HPO₄, and 15 g agar, with the final pH adjusted to 9.0. The protease activity assay medium consisted of 10 g skim milk, 5 g soy peptone, 15 g tryptone, 5 g NaCl, and 15 g agar, also adjusted to pH 9.0. The cellulase activity detection medium included 1.0 g peptone, 0.1 g CaCl₂·7H₂O, 5.0 g NaCl, and 9 g agar, with a final pH of 9.0.

A total of 270 isolates were screened for amylase, protease, and cellulase activity using the dot inoculation method. Strains were spotted onto specific assay media and incubated at 28°C for 3–4 days. Amylase activity was visualized by iodine staining, protease by clear zones on skim milk agar, and cellulase by Congo red staining, followed by NaCl destaining. Enzyme activity was semi-quantitatively assessed by calculating the ratio of the hydrolysis zone diameter (*R*) to the colony diameter (*r*); a ratio of *R*/*r* ≥ 2 was considered indicative of high enzymatic activity (Alshehri et al., 2025; Hankin and Anagnostakis, 1977; Sarethy et al., 2011).

## 3 Results

### 3.1 Analysis of bacterial community structures and diversity based on 16S rRNA gene sequencing

#### 3.1.1 Bacterial community structure

**Figure 1:**
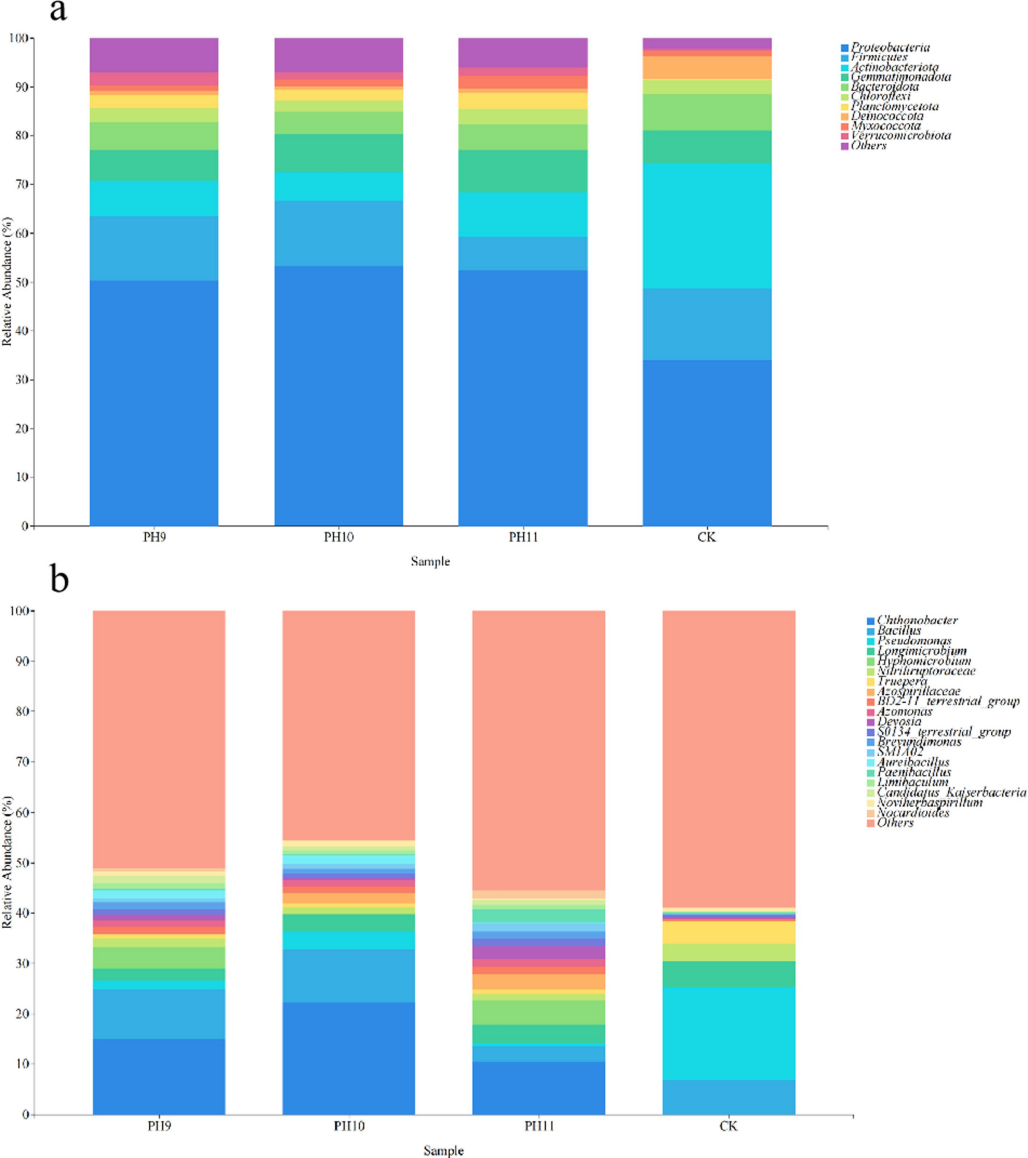
Community composition of bacterial groups under different culture conditions at the phylum level **(a)**. Community structure composition at the genus level **(b)**.

High-throughput sequencing yielded 2,433,549 original; 1,985,042 valid; and 10,393 ASVs. There was slight difference in the bacterial community structure between the different pH values, but there was a great difference in the community structure between the three groups and the untreated control group (Figure 1). The results of the no-culture analysis revealed 31 phyla and 207 genera in the pH 9 group. Proteobacteria, Firmicutes, and Actinobacteriota occupied the highest proportions (50.33, 13.16, and 7.25%, respectively). The dominant genera were *Chthonobacter*, *Bacillus*, and *Pseudomonas*, accounting for 14.97, 9.99, and 1.63% of all bacteria, respectively. A total of 32 phyla and 184 genera were detected in the pH 10 group. At the phylum level, Proteobacteria, Firmicutes, and Actinobacteriota were the most predominant, with proportions at 53.22, 13.49, and 5.83%, respectively. The dominant genera were *Chthonobacter*, *Bacillus*, and *Pseudomonas*, accounting for 22.30, 10.56 and 3.57%, respectively. Thirty phyla and 196 genera were detected in the pH 11 group. At the phylum level, Proteobacteria, Firmicutes, and Actinobacteriota occupied the highest proportions (52.32, 9.05, and 7.06%, respectively). The dominant genera were *Chthonobacter*, *Bacillus*, and *Pseudomonas*, accounting for 10.50, 2.98, and 0.71%, respectively. In total, 223 genera and 32 phyla were detected in the CK group. At the phylum level, Proteobacteria, Actinobacteriota, and Firmicutes accounted for the highest proportions (34.04, 25.47, and 14.76%, respectively). The dominant genera were *Bacillus*, *Pseudomonas*, and *Longimicrobium*, which accounted for 6.78, 18.54, and 5.19%, respectively.

#### 3.1.2 ASV number and α diversity index

**Figure 2:**
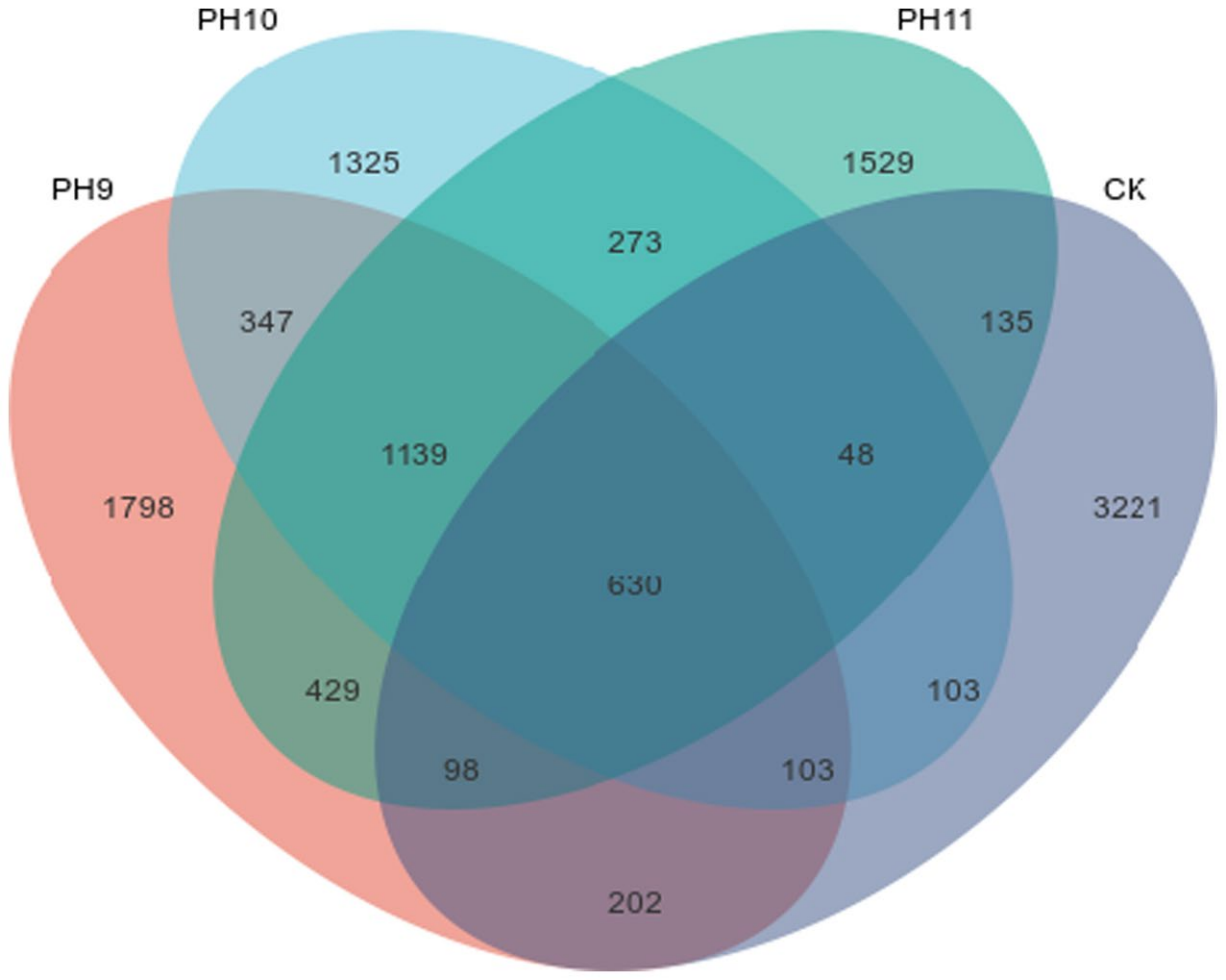
Venn diagram of the ASV quantities in the samples obtained from the Taklimakan Desert under different culture conditions.

This study investigated the diversity and abundance of microbial communities under different pH conditions (pH 9, pH 10, pH 11, and CK in the control group) (Table 2 and Figure 2).

**Table 2:** Comparison of alpha diversity indices under different culture conditions.

Medium	ASVs	Chao1 index	Shannon index	Simpson	Faith_pd	Observed_species	Pielou_e	Goods_coverage (%)
pH9	2,428	1198.1158 ± 590.4759a	7.0552 ± 1.7793a	0.9406 ± 0.0625a	73.9428 ± 28.7906a	1135.04 ± 557.7814a	0.6994 ± 1259a	99.69%
pH10	1955	994.4268 ± 127.4936a	6.4697 ± 1.8813a	0.9149 ± 0.0924a	67.3098 ± 26.189a	937.68 ± 480.231a	0.6582 ± 0.1399a	99.73%
pH11	2,159	1070.6222 ± 490.7391a	7.0721 ± 1.1531a	0.9571 ± 0.0223a	68.8436 ± 26.3593a	1013.82 ± 459.7121a	0.7153 ± 0.0683a	99.73%
Ck	3,851	1062.8774 ± 529.7809a	7.1731 ± 1.7758a	0.909 ± 0.1163a	73.7435 ± 29.3913a	1052.42 ± 528.3535a	0.7274 ± 0.156a	99.91%

Data are presented as the mean ± standard deviation. Different lowercase letters in the same column of data in the table indicate that the data at different dilution levels are significantly different (*p* < 0.05), and the same letter indicates that the data at different dilution levels are not significant (*p* > 0.05).

As shown in Table 2, the number of single sequence variants (ASVs) in the pH 9 samples was 2,428, which was significantly higher than that in the pH 10 (1955) and pH 11 (2159) samples, indicating that microbial community richness was highest in the pH 9 group. In addition, the CK samples had the highest number of ASVs (3851), indicating the diversity of microbial species under the control conditions. The Chao1 index in the pH 9 group was 1198.12 ± 590.4759, indicating that the expected richness of the microbial population under this condition was higher than that under other conditions. In terms of diversity assessment, the Shannon index at pH 9 was 7.05 ± 1.7793, indicating good species diversity, which was similar at pH 11 (7.07 ± 1.1531) and relatively low at pH 10 (6.47 ± 1.8813). In terms of the Simpson index, the value at pH 11 was 0.9571 ± 0.2236, which was the lowest among all conditions, indicating that the distribution of the microbial community under this condition was relatively uniform. The number of species observed was highest at pH 9, reaching 1135.04 ± 557.7814, suggesting that the community under pH 9 may be better adapted or more stable under alkaline conditions.

Figure 2 shows the ASVs shared among the different samples. The number of unique ASVs at pH 9 was 1798, indicating that a large number of unique microbial species existed under this pH. Although the total number of ASVs was the highest (3221) in the CK samples, the number of shared microbial species (273) was relatively small, suggesting that the microbial community composition under these conditions exhibited habitat-specific adaptation.

In summary, pH 9 fostered microbial communities with the highest overall abundance and diversity. Alpha-diversity analysis showed that the CK (untreated) soils contained the greatest number of observed species (1052.42 ± 528.35), reflecting high richness; however, their Shannon diversity and Pielou evenness values were lower than those at pH 9. Thus, while CK harbored more taxa, the community at pH 9 was more evenly distributed and therefore likely more competitively balanced. Together, these results underscore the pivotal influence of pH on shaping microbial community structure and sustaining soil biodiversity.

#### 3.1.3 Analysis of bacterial co-occurrence network under different pH conditions

**Figure 3:**
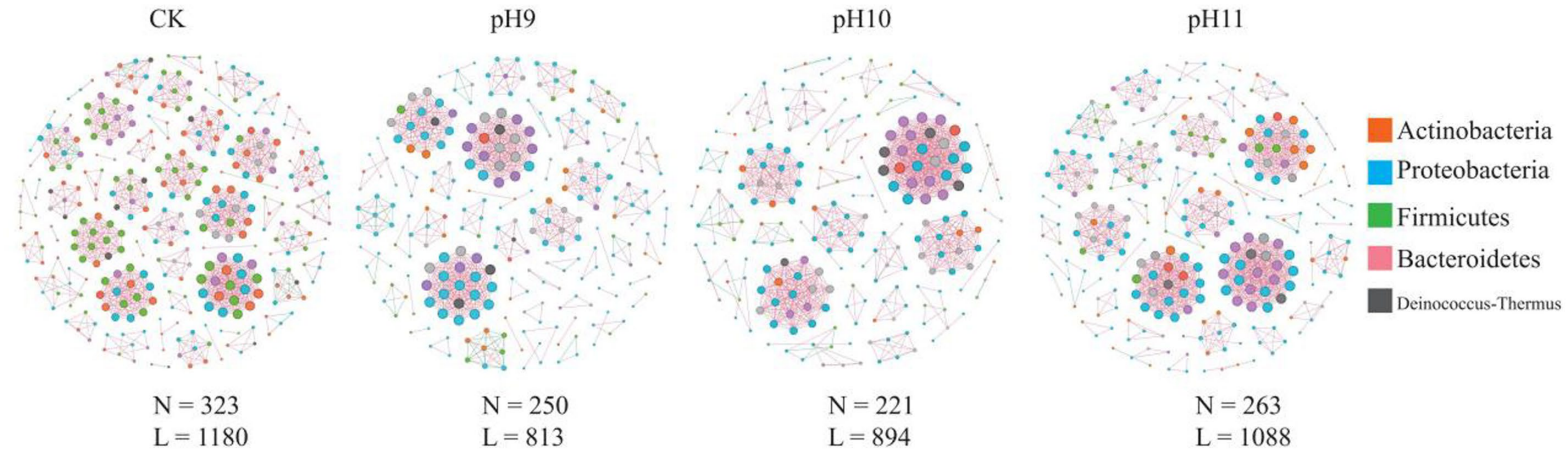
Bacterial co-occurrence networks under different pH treatments. N indicates the number of nodes in the network; that is, the number of different microbial species; L indicates the number of connected edges in the network, i.e., the number of interactions between microorganisms.

In this study, the structure and characteristics of microbial communities under different pH conditions (CK, pH 9, pH 10, and pH 11) were investigated using network analysis (Table 3 and Figure 3). The results showed that the number of nodes (*N* = 323) and edges (*L* = 1,180) were the highest in the CK environment, indicating high microbial diversity and a complex interaction network. With an increase in pH, the number of nodes and edges gradually decreased; *N* dropped to 250, and *L* dropped to 813 at pH 9, indicating that the interaction between microorganisms was weakened. At pH 10 (*N* = 221, *L* = 894) and pH 11 (*N* = 263, *L* = 1,088), although the number of microbial species recovered, the number of edges did not increase significantly, indicating the adaptability of specific microorganisms to high pH environments. The network analysis revealed that the average path length and network diameter of each environment was 1, indicating that the network had good connectivity. The clustering coefficients were all 1, indicating a close relationship between the nodes. The density (0.0227) and centralization (0.0332) of CK were the lowest, indicating that the distribution of microorganisms was relatively uniform, whereas the heterogeneity at pH 10 was the highest (0.9806), indicating that the difference between different microbial classes was enhanced. Overall, these findings revealed the profound impact of pH changes on microbial community structure and ecological networks, reflecting the dynamic characteristics of microbial communities and their ability to adapt to environmental changes.

**Table 3:** The topological index of the collinear network of microorganisms under different pH culture conditions.

	CK	pH9	pH10	pH11
Vertex	323	250	221	263
Edge	1,180	813	894	1,088
Average_degree	7.306501548	6.504	8.090497738	8.273764259
Average_path_length	1	1	1	1
Network_diameter	1	1	1	1
Clustering_coefficient	1	1	1	1
Density	0.022690999	0.026120482	0.03677499	0.031579253
Heterogeneity	0.662757935	0.899820781	0.980640728	0.868944923
Centralization	0.033209623	0.046168675	0.076861374	0.052390213
Modularity	0.939725654	0.884120133	0.813229134	0.875968115

### 3.2 Basophilic microorganisms strains found in the Taklimakan Desert

A large number of basophilic microbial strains were isolated and cultured from samples collected at different points in the Taklimakan Desert. The results of the 16S rRNA gene sequencing and sequence comparison analysis of the bacterial species is presented in Table 4. From the soil of the Taklimakan Desert, a total of 291 bacterial strains were isolated and identified, which belong to 56 genera, 25 families, 17 orders and 4 phyla. In total, 271 strains with evolutionary significance were identified. The dominant bacterial groups were Firmicutes and Actinobacteria. Bacilli and Actinomycetia were the dominant bacilli. Bacillales and Streptomycetales were dominant bacillales. *Bacillaceae* and *Streptomycetaceae* were dominant bacillaceae. The dominant bacteria identified were *Planococcus*, *Streptomyces,* and *Alkalihalobacillus*. Among them, 114 suspected new species showed less than 98.65% similarity.

**Table 4:** Classification of microorganisms present in the Taklimakan Desert obtained by isolation and culture.

Phylum	Class	Order	Family	Genus	Quantity
Actinobacteria	Actinomycetia	Cellulomonadales	*Promicromonosporaceae*	*Isoptericola*	1
		Geodermatophilales	*Geodermatophilaceae*	*Blastococcus*	1
		Microbacteriales	*Microbacteriaceae*	*Agrococcus*	1
				*Diaminobutyricimonas*	2
				*Microbacterium*	1
				*Pseudolysinimonas*	4
		Micrococcales	*Micrococcaceae*	*Arthrobacter*	10
				*Kocuria*	1
				*Micrococcus*	2
				*Nesterenkonia*	4
		Pseudonocardiales	*Pseudonocardiaceae*	*Stutzerimonas*	4
		Streptomycetales	*Streptomycetaceae*	*Streptomyces*	38
		Streptosporangiales	*Nocardiopsaceae*	*Nocardiopsis*	15
Bacteroidota	Cytophagia	Cytophagales	*Cyclobacteriaceae*	*Mongoliicoccus*	1
			*Hymenobacteraceae*	*Pontibacter*	2
				*Adhaeribacter*	2
Firmicutes	Bacilli	Bacillales	*Bacillaceae*	*Alkalihalobacillus*	23
				*Anaerobacillus*	4
				*Aquibacillus*	2
				*Bacillus*	14
				*Cytobacillus*	10
				*Domibacillus*	1
				*Evansella*	1
				*Fictibacillus*	2
				*Litchfieldia*	1
				*Oceanobacillus*	3
				*Ornithinibacillus*	6
				*Paucisalibacillus*	1
				*Peribacillus*	1
				*Pseudalkalibacillus*	1
				*Robertmurraya*	3
				*Rossellomorea*	2
				*Sediminibacillus*	1
				*Shouchella*	4
				*Sutcliffiella*	1
				*Mesobacillus*	2
				*Metabacillus*	18
			*Paenibacillaceae*	*Paenibacillus*	4
			*Planococcaceae*	*Metaplanococcus*	1
				*Planococcus*	64
Proteobacteria	Alphaproteobacteria	Rhizobiales	*Aurantimonadaceae*	*Aureimonas*	1
		Rhodobacterales	*Rhodobacteraceae*	*Paracoccus*	5
			*Phyllobacteriaceae*	*Chelativorans*	2
			*Devosiaceae*	*Pelagibacterium*	8
			*Salinarimonadaceae*	*Salinarimonas*	1
		Rhodospirillales	*Rhodovibrionaceae*	*Fodinicurvata*	2
			*Azospirillaceae*	*Indioceanicola*	1
			*Acetobacteraceae*	*Pseudoroseomonas*	1
		Sphingomonadales	*Erythrobacteraceae*	*Croceibacterium*	1
				*Erythrobacter*	1
	Betaproteobacteria	Burkholderiales	*Oxalobacteraceae*	*Noviherbaspirillum*	1
	Gammaproteobacteria	Lysobacterales	*Lysobacteraceae*	*Lysobacter*	4
				*Luteimonas*	1
		Oceanospirillales	*Halomonadaceae*	*Halomonas*	1
		Pseudomonadales	*Pseudomonadaceae*	*Pseudonocardia*	1
				*Saccharomonospora*	1

### 3.3 Number of colonies of the isolated species at different pHs and medium concentrations

**Figure 4:**
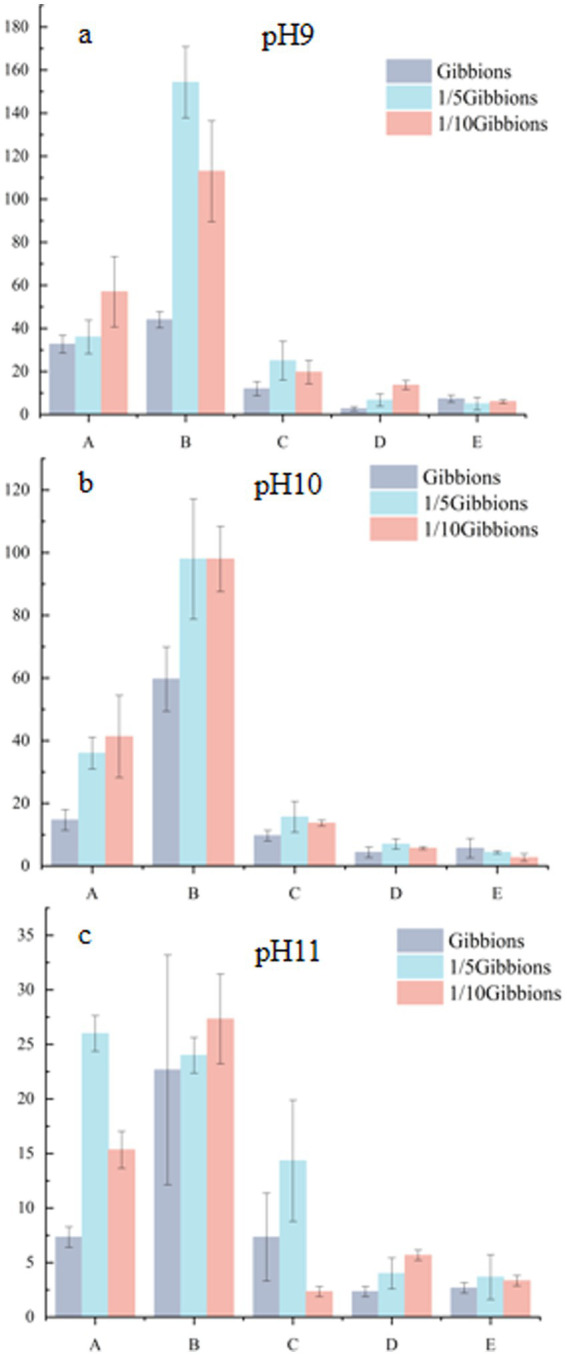
Number of colonies at different pHs and medium concentrations.

Strains obtained from the five soil samples collected from different latitudes and longitudes in the Taklimakan Desert were isolated, and plate counts of microbial colonies were performed by adjusting the pH values and medium concentrations (Figure 4). The overall trend showed that with an increase in the pH of the medium, the number of colonies on the plate gradually decreased. Although a decrease in colony number was observed with increasing medium pH, this pattern may also be influenced by factors such as the original soil pH (8.72–9.8), microbial viability, and nutrient conditions. The total number of colonies reached a maximum at pH 9. This indicates that the environmental conditions at pH 9 are the closest to the natural living environment of microorganisms in the soil of the Taklimakan Desert, which can more effectively promote bacterial growth and colony formation. In addition, the use of a highly alkaline medium helps isolate bacteria with strong alkali resistance. Among the tested media, the 1/10 Gibbons culture medium, due to its diluted nutrient content, better reflected the oligotrophic nature of the Taklimakan Desert soil. This low-nutrient condition effectively promoted the growth of microorganisms adapted to nutrient-poor environments, thereby improving colony separation efficiency. The use of low-nutrient media has been shown to facilitate the isolation of oligotrophic microbes by mimicking natural environmental constraints (Molina-Menor et al., 2020).

### 3.4 Influence of different pHs on the isolation culture

**Figure 5:**
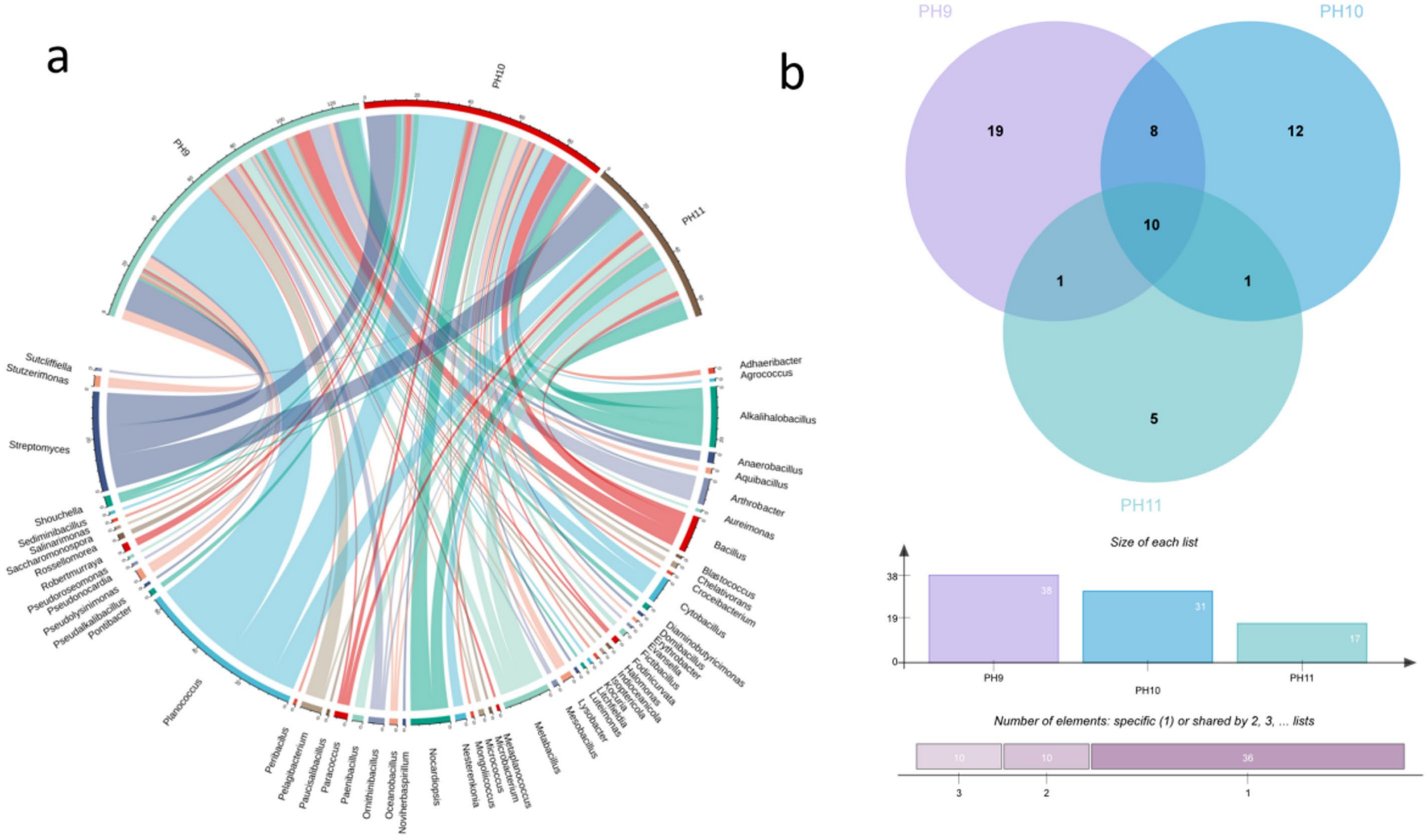
Microorganisms isolated from five soil samples in the Taklimakan Desert under different pH culture conditions. The string graph **(a)** of the bacterial community in the desert samples represents the differences in the distribution of microorganisms in the Taklimakan Desert soil samples under different pH culture conditions. The Venn diagrams of bacterial communities in desert samples under different pH culture conditions represent the quantity distribution of genus **(b)**. These samples are represented by different circles: the Venn diagram shows the number of genera detected in each sample and the overlap of genera in the sample.

Microorganisms present in the Taklimakan Desert were isolated and cultured under different pH gradient media conditions, and the diversity of the microorganisms obtained at the genus level is shown in Figure 5. At pH 9, 130 strains were isolated and were distributed across 38 genera. At pH 10, 95 strains were isolated and distributed across 37 genera. At pH 11, 66 strains were isolated and were distributed across 17 genera. The specific strains at pH 9 were *Agrococcus*, *Aquibacillus*, *Blastococcus*, *Chelativorans*, *Croceibacterium*, *Diaminobutyricimonas*, *Evansella*, *Halomonas*, *Indioceanicola*, *Kocuria*, *Litchfieldia*, *Metaplanococcus*, *Peribacillus*, *Pseudalkalibacillus*, *Pseudolysinimonas*, *Pseudoroseomonas*, *Saccharomonospora*, *Salinarimonas*, and *Stutzerimonas*. The pH 10 endemic bacteria were *Adhaeribacter*, *Domibacillus*, *Erythrobacter*, *Fictibacillus*, *Isoptericola*, *Luteimonas*, *Microbacterium*, *Noviherbaspirillum*, *Paucisalibacillus*, *Pontibacter*, *Pseudonocardia*, and *Sediminibacillus* and pH 11 was endemic to *Aureimonas*, *Mongoliicoccus*, *Nesterenkonia*, *Paenibacillus*, and *Sutcliffiella*. *Alkalihalobacillus*, *Arthrobacter*, *Bacillus*, *Metabacillus*, *Oceanobacillus*, *Ornithinibacillus*, *Paracoccus*, *Planococcus*, *Shouchella*, and *Streptomyces* were found at all three pH conditions.

### 3.5 Influence of medium dilution concentration on microbial isolation and culture

**Figure 6:**
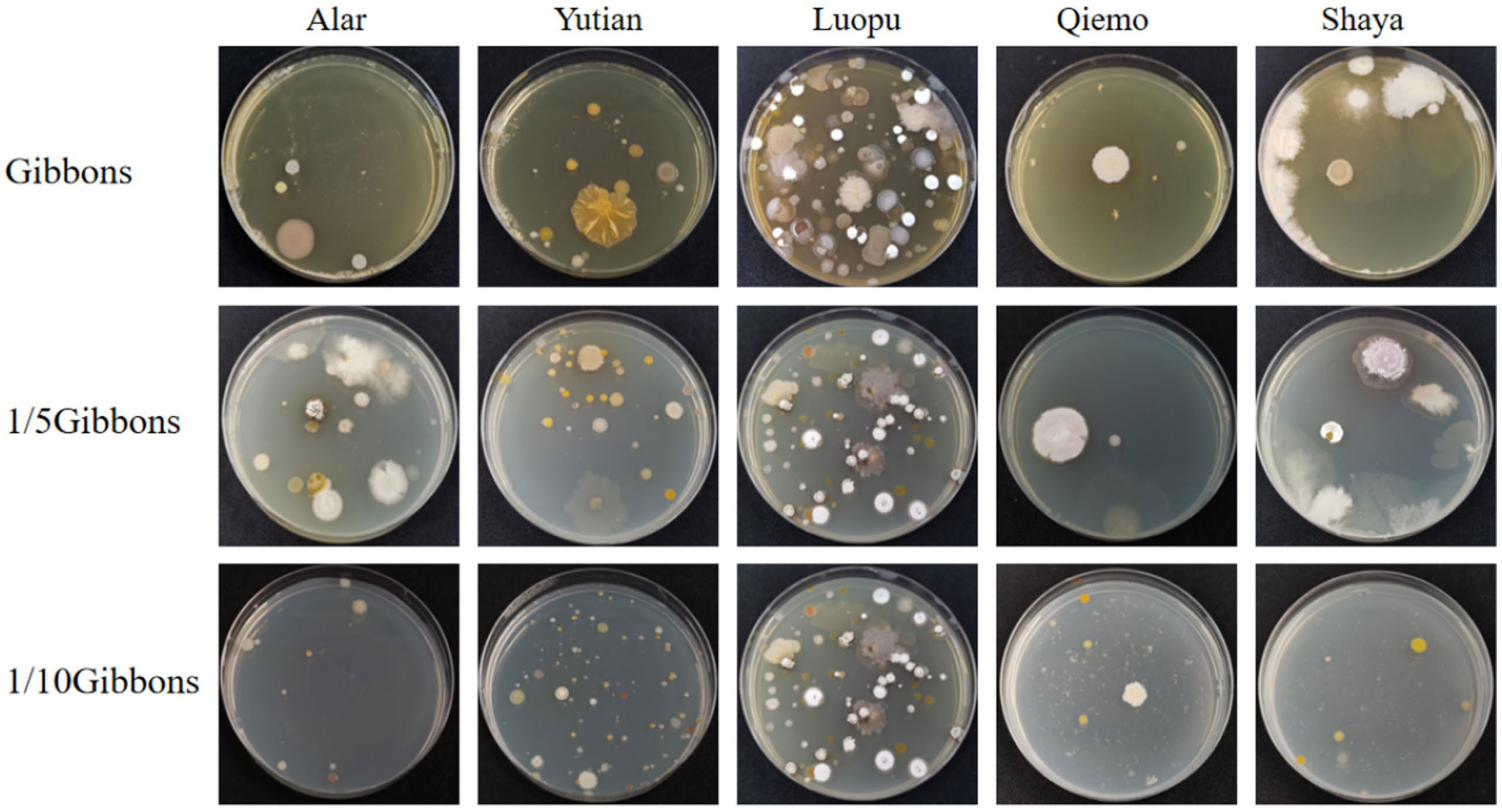
The colony formation of five soil samples on Gibbons medium with different dilution concentrations.

Gibbons culture medium was used at final concentrations of 1, 1/5 and 1/10. Overall, colony growth was optimal at a dilution concentration of 1/10; the morphology was clear, and the species abundance was the highest. The bacterial growth of the five soil samples on Gibbons culture medium for each dilution concentration gradient is shown in Figure 6.

### 3.6 Distribution of bacteria obtained from media with different dilution concentrations

**Figure 7:**
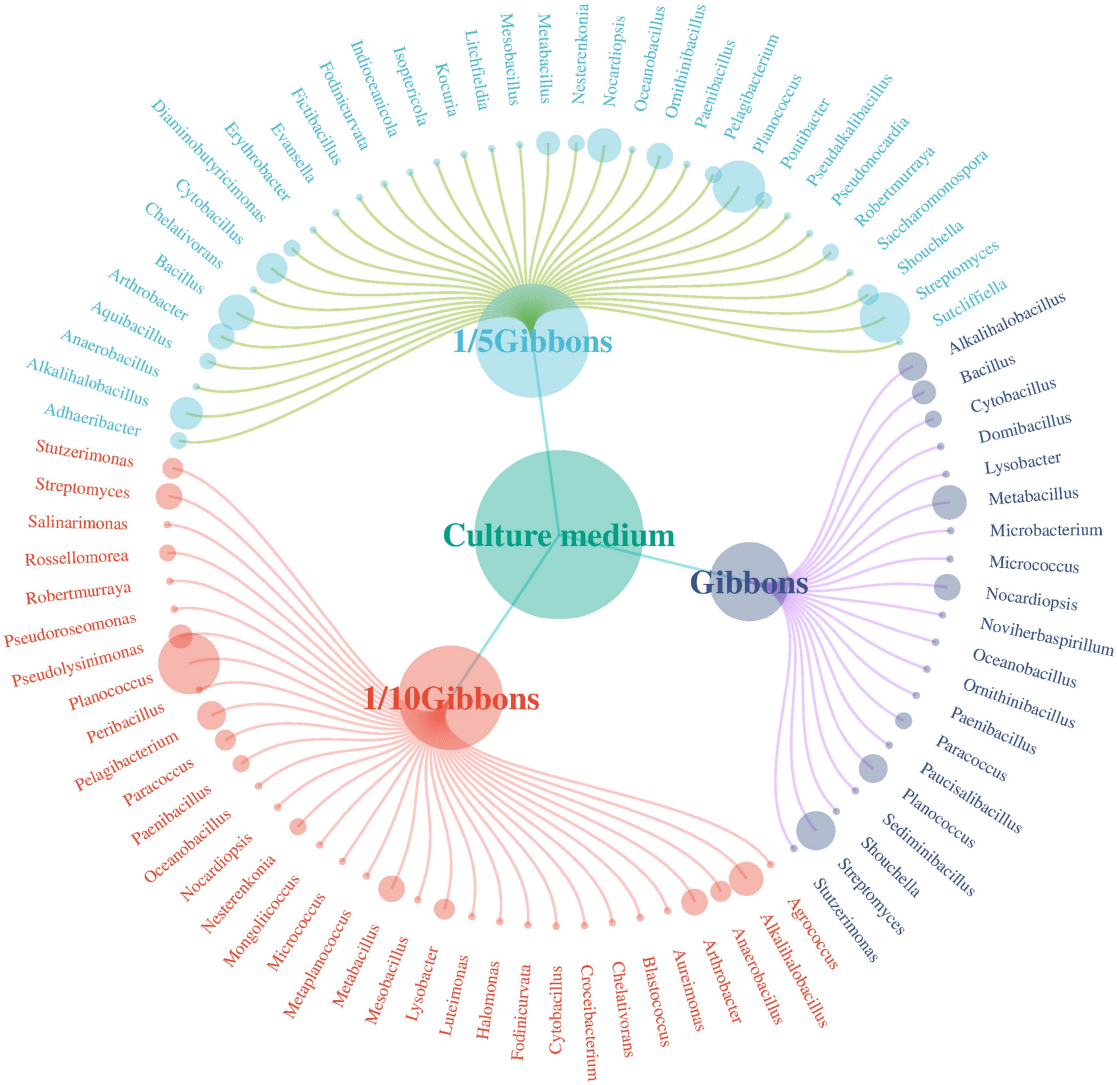
Distribution of bacterial species obtained from Gibbons media at different dilution concentrations.

Basophilic microorganisms were isolated from soil samples obtained from the Taklimakan Desert and cultured under different medium concentrations, and the diversity of microorganisms obtained at the genus level is shown in Figure 7.

A total of 291 species belonging to 56 genera were obtained from the Gibbons cultures. Fifty-nine strains were isolated from the undiluted Gibbons medium, distributed across 21 genera, including five genera that were only detected under this condition: *Domibacillus*, *Microbacterium*, *Noviherbaspirillum*, *Paucisalibacillus*, and *Sediminibacillus*. One hundred twenty-eight strains were isolated from the 1/5 Gibbons medium, spanning 34 genera, with 15 genera found exclusively in this condition: *Adhaeribacter*, *Diaminobutyricimonas*, *Erythrobacter*, *Evansella*, *Fictibacillus*, *Indioceanicola*, *Isoptericola*, *Kocuria*, *Litchfieldia*, *Ornithinibacillus*, *Pontibacter*, *Pseudalkalibacillus*, *Pseudonocardia*, *Saccharomonospora*, and *Sutcliffiella*. One hundred four strains were obtained from the 1/10 Gibbons medium, affiliated with 33 genera, including 12 that were uniquely observed under this condition: *Agrococcus*, *Aureimonas*, *Blastococcus*, *Croceibacterium*, *Halomonas*, *Luteimonas*, *Metaplanococcus*, *Mongoliicoccus*, *Peribacillus*, *Pseudolysinimonas*, *Pseudoroseomonas*, and *Salinarimonas*. However, given that all isolates originated from dilutions of the same composite sample, and the number of strains per condition was limited, the presence of these genera under specific culture conditions likely reflects differential recovery efficiency rather than true habitat specificity or endemism.

### 3.7 Screening of saline-alkali resistant strains and determination of saline-alkali tolerance

A total of 271 phylogenetically representative strains were selected for tolerance tests at pH 7–12 and salinities of 0–25%.

#### 3.7.1 Salt tolerance of each strain

The salt tolerance of the strains was compared by gradually increasing the salt concentration in a medium at pH 9 to determine whether they were able to grow (Supplementary Table S1). The results showed that 247 strains could tolerate 5% salt concentration and 162 strains could tolerate 0 to 15% salt concentration, indicating strong salt tolerance. Additionally, 121 strains tolerated 0 to 25% salt concentration, indicating that they are high-salt-tolerant microorganisms. These results indicate that there are many halophilic and salt-tolerant species among the microbial communities in the soil of the Taklimakan Desert, which provides a basis for studying their functional characteristics.

#### 3.7.2 Alkali resistance of each strain

At 5% salt concentration, the pH of the medium was gradually increased to assess the growth of each strain (Supplementary Table S1). We found that 51 strains had weak alkali resistance and could not grow at pH 11.0, and 71 strains could not tolerate pH 12.0. A total of 166 strains grew at pH 7–12 and were considered to be alkali-resistant microorganisms. Among these, 38 strains could only grow in the pH 9–12 range and were considered basophilic microorganisms. In addition, 85 strains could not only grow at pH 12 but could also tolerate 25% salt, accounting for 31.48% of the total strains. These strains were extremely resistant to salt and alkali stress and exhibit unique characteristics that allow them to adapt to extreme environments.

#### 3.7.3 Alkali resistance of each strain

With the culture medium at pH 12, the tested strains were cultured with 15 and 25% salinity; 34 strains tolerated pH 12 and 15% salinity, and 32 strains tolerated pH 12 and 25% salinity, but the growth of the strains was weak (Supplementary Table S2).

### 3.8 Functional enzyme productivity of basophilic microorganisms

**Figure 8:**
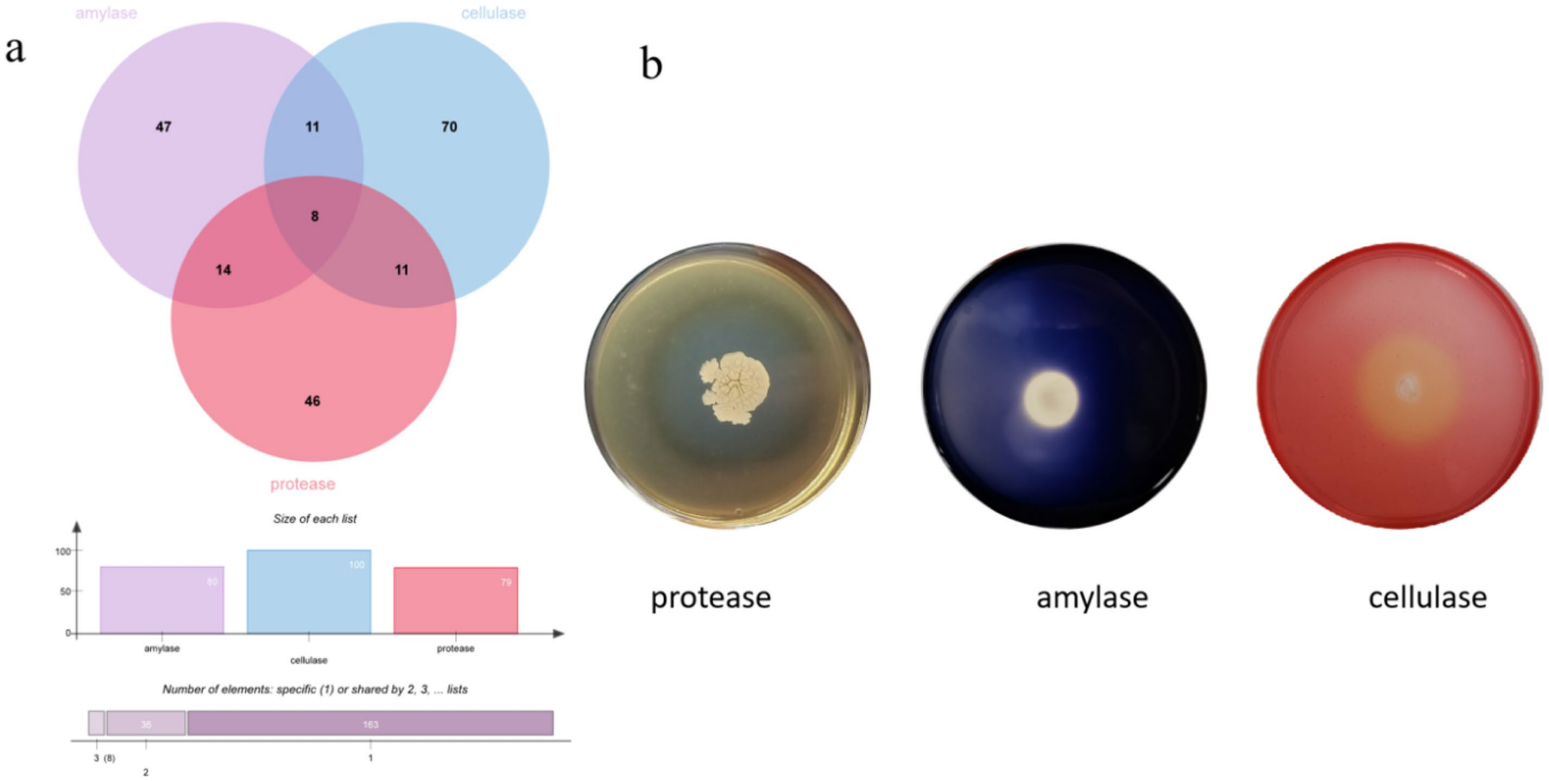
Amylase, protease, and cellulase activity screening, displayed using a number Wayne map **(a)** and functional enzyme screening map for some strains **(b)**.

**Figure 9:**
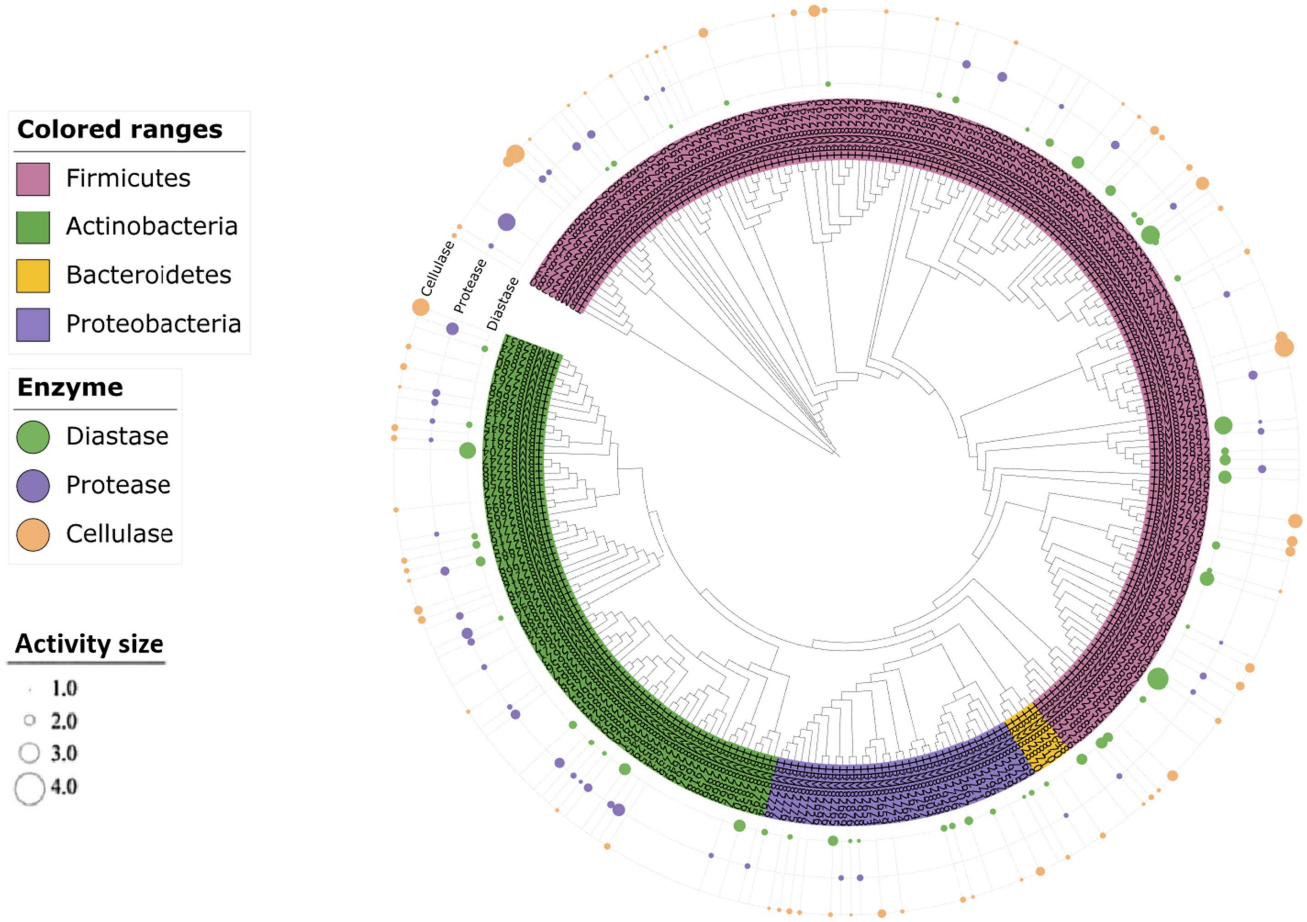
Clustering analysis of the hydrolytic properties of different substrates produced by the strains. The size of the dots indicates the level of the hydrolysis activity of the substrate.

The enzyme production capacities of 271 isolates were screened, and it was found that 47 strains produced amylase, 46 strains produced protease, and 70 strains produced cellulase, accounting for 20.35, 19.91, and 30.30% of all the strains, respectively. At least one functional enzyme was observed in 47.61% of the isolates as shown in Figures 8, 9. The predominant amylase-producing bacteria were *Streptomyces*, *Planococcus*, *Metabacillus*, and *Bacillus*. The predominant proteinase-producing bacteria were *Streptomyces*, *Planococcus*, *Bacillus* spp., and *Nocardiopsis*. The predominant cellulose-producing bacteria were *Planococcus*, *Metabacillus*, *Stutzerimonas,* and *Nocardiopsis*. The discovery of multi-enzyme-active strains indicates that the microbial community in the soil of the Taklimakan Desert has formed diverse metabolic mechanisms over time, and screening of its enzyme activities may be significant. The stability and high efficiency of these enzymes have significant application potential in the detergent, food processing, and textile industries.

## 4 Discussion

### 4.1 Distribution characteristics and application potential of alkalophilic bacteria present in the desert

The diversity of alkali-tolerant bacteria found in the Taklimakan Desert was analyzed using culture-independent and pure culture methods. Four sets of high-throughput sequencing data showed that the dominant bacterial groups in this desert were Proteobacteria, Actinobacteriota, and Firmicutes; similar results were obtained using pure cultures. Sorokin et al. (2017) analyzed saline-alkali soil samples with pH > 9 from eight locations worldwide and reported that Actinobacteria and Firmicutes were the dominant bacterial groups, which aligns with the results of this study. After alkaline buffer solution treatment, high-throughput sequencing results showed that the dominant bacteria, Proteobacteria, Firmicutes, Actinobacteria, and Firmicutes, were more abundant than their corresponding bacteria in group CK. This phenomenon may be related to the alkaline resistance and adaptability of these bacteria. Proteobacteria was the most abundant group, and their diverse metabolic functions, including involvement in nutrient cycling and stress response, suggest they play a key ecological role in the desert soil environment (Ren et al., 2018). Second, Actinobacteria showed good adaptability to high temperature, high salinity, and strong radiation, which may be related to their ability to produce spores with strong stress resistance, extensive metabolic capacity, competition from secondary metabolic anti-bioactive substances, and the ability to repair UV damage (Bull and Asenjo, 2013). Firmicutes can withstand adverse conditions through spore production. Bacterial spores in a dormant state can survive harsh environments and can be revived under suitable conditions. This dormant state allows the bacteria to survive and spread under harsh conditions.

In this study, the isolation and characterization of alkaliphilic microorganisms revealed that *Planococcus* was the dominant genus following alkali treatment. This result contrasts with Wen et al. (2024), who used media with varying nutrient levels and identified *Streptomyces* as the predominant genus in the Taklimakan Desert. The difference may be attributed to the selective pressure of alkaline conditions in our study, which favored the enrichment of haloalkaliphilic taxa such as *Planococcu*s, rather than nutrient-responsive actinomycetes like *Streptomyces*. In previous studies, *Planococcus* were found to be the dominant species in many marine environments, such as the deep sea, salt marshes, and intertidal zone (Engelhardt et al., 2001; Jung-Hoon et al., 2010; Yoon et al., 2003). However, the results of this study indicate that *Planococcus* also shows strong adaptability to alkaline conditions, exhibiting significantly higher abundance than other genera under alkali-treated conditions. While *Planococcus* has previously been isolated from cold and saline environments such as the Arctic, Antarctic, and marine ecosystems (Jung-Hoon et al., 2010; Reddy et al., 2002; Mykytczuk et al., 2012), these habitats often feature both high salinity and alkaline pH. This co-occurrence suggests that *Planococcus* may possess adaptive mechanisms enabling it to tolerate both salt and alkaline stress. For example, recent studies have isolated alkaliphilic *Planococcus* strains from saline–alkaline soils in northwestern China, reinforcing its ecological flexibility (Jin et al., 2024). Our findings extend this understanding by demonstrating that *Planococcus* thrives under alkaline culture conditions and exhibits notable hydrolytic enzyme activity. In particular, the strain isolated in this study produced all three tested enzymes, supporting its previously reported functional roles in cold-adapted protease production (Chen et al., 2018; Leng et al., 2021, 2023). In addition, *Planococcus* strains can produce carotenoids, which can be used in cosmetics, food, or feed additives, and as functional components of antioxidants (Lee and Schmidt-Dannert, 2002). *Planococcus* also has significant advantages in environmental pollution control; it has been proven to degrade and treat a variety of pollutants and can be used for biological remediation in extreme environments (Hupertkocurek et al., 2012; Sun et al., 2014).

In this study, analysis of the unique bacterium *Halomonas* isolated at pH 9 revealed consistencies with the results of previous studies, which showed that *Halomonas* was the most abundant gram-negative bacterium in soda lakes and other high-salt environments (Simachew et al., 2016) and could grow on nutrient-poor media. In this study, we successfully isolated a member of this genus in a 1/10 dilution Gibbons medium and demonstrated its potential to produce amylase and cellulase. This suggests that the *Halomonas* genus is highly adaptable and can survive and reproduce under resource-limited conditions. In addition, some members of this genus possess diazotrophic characteristics (Llamas et al., 2006), indicating a possible mechanism for their survival in extreme environments. *Mongoliicoccus* was uniquely isolated at pH 11 and was not recovered from any other pH conditions. Currently, only two alkalophilic bacteria strains have been isolated from saline-alkali lakes (Liu et al., 2012; Subhash et al., 2013). However, to the best of our knowledge, this is the first study to isolate *Mongoliicoccus* from arid alkaline desert soils. This not only expands the ecological distribution range of this genus but also further demonstrates that this genus is highly adaptable to extreme environments. The shifts in bacterial genera across different pH treatments (from neutral to highly alkaline) reflect the differential adaptability of desert microorganisms to varying pH conditions. This pH-driven selection provides a valuable foundation for future studies exploring the functional traits of alkaliphilic microbes and their potential applications in extreme environments.

A total of 291 strains were isolated, and 114 exhibited low similarity to known type strains, suggesting they may represent potential new taxa pending further taxonomic validation. This discovery not only provides new insights into the classification and phylogeny of microorganisms but also further enriches the gene pool of microbial species, especially in the study of microbial diversity in extremely alkaline environments.

### 4.2 Influence of culture conditions on microbial isolation silk-velvet replicate

The efficiency of microbial isolation methods positively correlates with the selection of the required media, supplementary inhibitors, inoculation techniques, and culture conditions that simulate desert environments (Goodfellow and Fiedler, 2010; Tiwari and Gupta, 2013; Zhang et al., 2014). Li et al. (2021) showed that most desert actinomycetes are slow-growing actinomycetes, preferring nutrient-poor media to nutrient-rich media. In this study, we compared the effects of different pHs and medium dilutions on microbial isolation. The results showed that pH 9 and a dilution concentration of 1/10 were the best conditions for isolation, as the highest diversity of strains was obtained under these conditions. This shows that in an alkaline desert environment, the isolation effect of microorganisms is not only greatly affected by the pH value of the medium but is also closely related to the nutrient concentration of the medium.

Microbes in deserts prefer to grow in low-water environments. Compared to the traditional continuous dilution method, Xie was able to isolate more strains using sprinkler inoculation technology, and a higher bacterial diversity was recovered from mineral particles on selective medium supplemented with inhibitors using the sprinkler technology (Idris et al., 2017). In contrast to media inoculated with soil suspensions, this study applied the silk-velvet replicate technique, in which the inoculation process does not involve water and allows mineral particles to come into direct contact with the nutrients in the medium. This condition may stimulate spore germination and enhance the growth of bacteria and actinomycetes. Our previous studies using the silk-velvet replicate technique in combination with different nutrient levels to isolate microorganisms from the Taklimakan Desert also yielded an untapped diversity of culturable bacteria; therefore, we strongly recommend the use of silk-velvet replicate technique to isolate bacteria from desert-related samples.

It is important to note that the microbial community profiles obtained in this study were based on DNA extracted from enrichment cultures, rather than directly from the original soil samples. While this approach enabled us to explore the diversity of alkali-tolerant and fast-growing bacteria under specific pH and nutrient conditions, it inevitably introduced a selection bias favoring cultivable and metabolically active taxa under laboratory settings. Therefore, the results do not fully capture the total microbial diversity present *in situ*, and conclusions regarding environmental diversity should be interpreted with caution. To address this limitation, we also included a control group (CK) composed of pooled original soil samples without enrichment treatment, allowing for a baseline comparison of the native bacterial community.

### 4.3 Screening and functional characteristics of saline-alkali resistant microorganisms

In this study, a group of saline alkali-resistant strains were selected from the soil of the Taklimakan Desert through isolation, culture, and functional screening. These strains show strong environmental adaptability and provide a scientific basis for exploring potential applications of microbial resources in extreme environments. The results showed that 175 strains were able to grow at pH 12, and 78 strains could tolerate a salt concentration of 25%. The viability of these strains suggests that they not only adapt to the extreme environment of the Taklimakan Desert but may also have developed special physiological mechanisms to cope with the high salt and alkali conditions in extreme environments, for example, by producing resistant extracellular enzymes, protective metabolites, or specific proteins that stabilize the cell structure (Gallo and Aulitto, 2024; Zhu et al., 2020).

Further functional screening showed that 47.61% of the isolates had functional enzyme activity, 20.35% had amylase activity, 19.91% had protease activity, and 30.30% showed cellulase activity. These strains were distributed across multiple phylogenetic clades, reflecting the genetic diversity and adaptation mechanisms of enzymatically active microorganisms (Raddadi et al., 2015) and indicating that desert microorganisms have evolved diverse enzyme-producing mechanisms and rich enzyme resources during long-term adaptation. Enzymes derived from basophilic substances have high thermal stability, alkaline activity, and substrate specificity, which are advantageous for biofuel production under harsh industrial conditions (Annamalai et al., 2016). In particular, strains with multifunctional enzyme activities have great potential for industrial applications. From an ecological perspective, these saline tolerant strains may regulate the alkalinity or salinity of the surrounding environment by secreting specific enzymes to maintain their survival advantages. Salt-tolerant microorganisms can adapt to high-salt environments by synthesizing protective osmoregulatory substances (such as betaine and glycerin) to reduce osmotic pressure differences inside and outside the cell (Roberts, 2005). Similarly, alkali-tolerant microorganisms maintain the acid–base balance in cells by regulating the activity of extracellular enzymes or the stability of cell membranes (Horikoshi, 1999).

This study not only reveals the diversity of microorganisms and their adaptation mechanisms in the Taklimakan Desert but also provides a foundation for the development of saline-tolerant microorganisms in extreme environments. The ability of these strains to tolerate high salt and alkali concentrations in industrial production means that they can maintain efficient and stable enzyme activity under extreme conditions that present difficulties when using traditional industrial enzyme preparations, thereby reducing production costs and improving efficiency. For example, the stability of saline-resistant alkalases in textile and detergent industries makes them important candidates for enzyme preparation (Katsimpouras and Stephanopoulos, 2021). In addition, these strains may also have potential applications in bioreactors, such as the ecological treatment of saline-alkali land and the degradation of extreme environmental pollutants (Bahram et al., 2018).

There are some limitations in this study that should be addressed in future research. First, although our sequencing approach targeted the bacterial communities enriched under alkaline conditions, it is still possible that DNA from non-growing or dead cells remained in the samples and contributed to the observed community profiles. Since no autoclaved soil inoculum was included as a blank control, we cannot fully exclude the influence of relic DNA. Future studies should incorporate sterile soil inoculation controls to better differentiate active microbial populations from background signals. Second, the use of pure culture methods inevitably restricts the discovery of certain microorganisms, particularly those that are difficult to cultivate under standard laboratory conditions. For example, the genus *Diaminobutyricimonas* was isolated in this study but was not detected in the culture-independent sequencing data, highlighting the complementary but limited scope of each method. In addition, the functional screening was confined to three enzymes—amylase, protease, and cellulase—which provides only a partial view of the potential industrial applications of these microorganisms. Future efforts should expand the enzyme repertoire to include lipases, xylanases, and other biocatalysts of industrial interest. While our findings suggest a decreasing trend in bacterial diversity and colony numbers under increasing alkaline conditions, this pattern should not be interpreted as being driven solely by pH. The complex interplay of other environmental variables—such as the original physicochemical properties of the soils, nutrient availability, and microbial viability—may also contribute to the observed community shifts. Therefore, future studies should aim to disentangle these confounding factors through improved experimental designs and comprehensive environmental profiling.

Nevertheless, this study provides an important basis for the study of microorganisms in the Taklimakan Desert, which can be combined with multi-omics techniques and molecular biology methods to further explore the dynamic changes and application potential of desert microorganisms in the future.

## 5 Conclusion

This study systematically explored the diversity of alkali-tolerant microorganisms and their potential applications in the Taklimakan Desert. High-throughput sequencing and pure culture techniques revealed that the community structure of alkali-tolerant bacteria in the soil samples differed under different pH conditions. A total of 291 bacterial strains were isolated using pure culture, among which 114 potential new taxa exhibited less than 98.65% similarity, revealing the rich microbial diversity in the desert soil. Further studies showed that these strains exhibited strong saline-alkali tolerance, with 85 strains able to grow at pH 12 and tolerate 25% salt concentrations. In addition, 47.61% of the strains had at least one functional enzyme activity, including amylase, protease, and cellulase, which provides a potential strain resource for the development of enzyme preparations for industrial applications. This study not only enriches the resource and gene pools of basophilic microorganisms in China but also provides a scientific basis for the exploitation and utilization of basophilic microorganism resources and the mining of stress resistance genes, which has important theoretical significance and practical application value.

## Data Availability

The datasets generated for this study can be found in the NCBI GenBank (https://www.ncbi.nlm.nih.gov/genbank/), under the accession numbers PQ825971-PQ826240.

## Footnotes

^1^ http://www.personalbio.cn

^2^ https://www.arb-silva.de

^3^ https://www.ezbiocloud.net
